# Geometric and Optic Characterization of a Hemispherical Dome Port for Underwater Photogrammetry

**DOI:** 10.3390/s16010048

**Published:** 2016-01-02

**Authors:** Fabio Menna, Erica Nocerino, Francesco Fassi, Fabio Remondino

**Affiliations:** 1 3D Optical Metrology unit, Bruno Kessler Foundation (FBK), via Sommarive 18, Trento 38123, Italy; nocerino@fbk.eu (E.N.); remondino@fbk.eu (F.R.); 2 Politecnico di Milano, ABC Dep. 3DSurvey Group, via Ponzio 31, Milano 20133, Italy; francesco.fassi@polimi.it

**Keywords:** underwater, photogrammetry, camera calibration, depth of field

## Abstract

The popularity of automatic photogrammetric techniques has promoted many experiments in underwater scenarios leading to quite impressive visual results, even by non-experts. Despite these achievements, a deep understanding of camera and lens behaviors as well as optical phenomena involved in underwater operations is fundamental to better plan field campaigns and anticipate the achievable results. The paper presents a geometric investigation of a consumer grade underwater camera housing, manufactured by NiMAR and equipped with a 7′′ dome port. After a review of flat and dome ports, the work analyzes, using simulations and real experiments, the main optical phenomena involved when operating a camera underwater. Specific aspects which deal with photogrammetric acquisitions are considered with some tests in laboratory and in a swimming pool. Results and considerations are shown and commented.

## 1. Introduction

Despite being a hostile environment both for humans and optical equipment, underwater measurement using photogrammetry can be feasible in several cases. Photogrammetry still represents the most useful recording technique currently available underwater due to its flexibility and ease of image data interpretation.

Nowadays the increasing number of demanding applications is growing constantly, mainly thanks to improvements of data processing software as well as technical achievements in diving apparatus, photographic equipment and underwater manned and unmanned vehicles. Furthermore, the widespread availability of scuba diving centers has expanded the knowledge and education about underwater environment even if only at a recreational level.

The general knowledge about underwater exploration and documentation has rapidly grown in the last few years and different users are demanding low-cost, quick, easy and fast 3D measurement solutions. Divers have developed the ability of taking notes and sketches underwater or acquiring photographs and videos. The popularity of automatic photogrammetric and structure from motion techniques in air among non-experts has stirred and also promoted experiments underwater, achieving impressive visual results [1,2,3] as much as serious concerns about safety. Indeed, appropriate training for in-water activities is crucial in order to assure safety. For this reason, diving courses for scientists who want to learn and safely practice digital recording techniques underwater have been proposed [4].

Since the beginning of underwater photography in 1850s by the pioneer William Bauer, it was noticeably obvious that the acquisition of underwater photographs would need severe modifications to photographic equipment. Nowadays, the causes of underwater photography problems are still often obscure to most users, and this is even more the case for non-experts in surveying and photogrammetry. In many projects, when conditions which guarantee safety for divers involved in the project are met, consumer cameras in their own underwater camera housings equipped with external strobe lights can be operated by divers.

Testing and investigating the geometrical characteristics of underwater consumer grade photographic equipment when used for photogrammetric applications would be advisable if accuracy and reliability matter. Professional results always rely on the control of all the technical parameters involved. The knowledge about photographic equipment and its behavior in different conditions is the first step to be investigated.

Whether they are in shallow or deep water, most underwater photogrammetric applications have to deal with a challenging optical environment due to water ripple reflections, light absorption and turbidity. Water is a medium inherently different from air and the first essential difference resides in the medium density. Seawater is nearly 800 times denser than air, and this influences the image formation underwater as the path of optical rays is altered. Density of seawater is not constant through depth, being a function of temperature, salinity and pressure. Although pressure is an extremely critical factor for very deep underwater inspections, its variation with depth affects any underwater optical system at whatever depth. Internal arrangement may be altered and subject to changes as the working depth varies.

By considering all the aforementioned constraining factors, underwater photogrammetry deals most of the time with close- and very close-range distances, usually maximum in the order of few meters. As for all close-range photogrammetric surveys carried out in air, the key parameters for network planning and acquisition—such as ground sample distance (GSD), baseline, image overlap, expected accuracy, nominal focal length, sensor resolution, aperture value, depth of field, *etc.*—must be known to plan the survey.

### Paper Aims, Methods and Tests

In this contribution a geometric investigation of a consumer grade underwater camera housing equipped with a 7′′ dome port is presented. The housing is specifically manufactured by NiMAR (Modena, Italy [5]) for a Nikon D300 camera. The system, composed by digital camera, lens, camera housing and dome port was already used by the authors for the underwater and in-air joint survey of the Costa Concordia ship gash [6].

The NiMAR NI303D is a pressure housing for the D300 camera (Nikon, Tokyo, Japan). It is made of polycarbonate and gives access to the most used buttons and functions of the camera. Through a bayonet at the front of the housing different ports, spherical and flat, can be mounted. As for many other housings made by other manufacturers, not all the camera functions are available due to the complexity in reaching levers or buttons over the camera body. Some missing functions are not a big problem most of the time, while some others may be a limitation for photogrammetric applications and make the operations more complex or longer to find proper workaround solutions.

The aim of the work is to analyze, using photogrammetric methods, the main optical phenomena which involve a camera operating underwater. Specific aspects which deal with photogrammetric acquisitions are considered and practical suggestions provided. Within the presented investigation, carried out with the Italian manufacturer of waterproof camera housings NiMAR, experimental setups are being designed and investigated to calibrate and test underwater camera housings for photogrammetric applications. Both simulations and tests underwater were carried out to design and implement systematic tests for underwater camera housings. Theoretical graphs and optical calculations for the underwater ports, both flat and dome are derived using the freely available WinLens 3D Basic and Predesigner and software application by Qioptiq (Goettingen, Germany [7]). Optical distortions for the camera-lens and underwater case-port system are based on formulas well known in optics and photogrammetry and coded by the authors in Matlab. Underwater acquisitions were performed in a 2 meter deep swimming pool.

## 2. Consumer Grade Flat and Dome Ports

**Figure 1:**
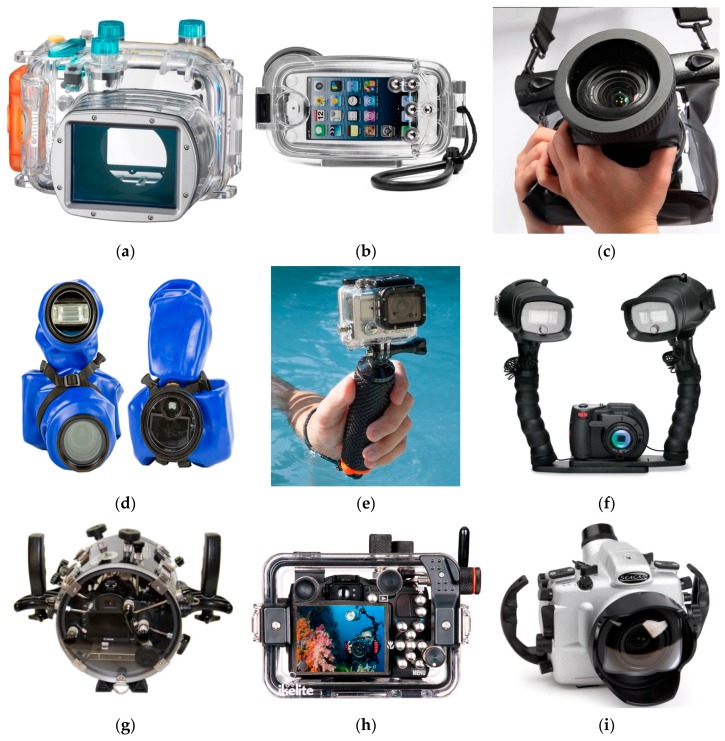
Waterproof housings for digital cameras and flashes: (**a**) Canon with flat port for compact cameras; (**b**) Watershot iPhone housing; (**c**) TteooBL waterproof bag; (**d**) Outex; (**e**) GoPro Hero 3 in its protective waterproof case (Christography); (**f**) Sealife with twin-flash (Hunteroc); (**g**) Equinox Housings for DSLR Nikon; (**h**) Ikelite for Canon; (**i**) Seacam with dome port.

Two types of lens port are employed in waterproof housing for underwater photography: flat and dome. Figure 1 features a variety of functional, fancy, professional and consumer-grade waterproof housings available on the market for any type of digital cameras and needs. Very low-cost consumer grade housings, such as the models shown in Figure 1b,c, can be made of non-rigid materials and consequently may be not well suited for photogrammetric acquisitions. Indeed, relative movements between the port and camera are likely to occur as pressure, temperature and even diver skills can continuously vary. The uncontrolled relative movements may cause optical distortions that are difficult to model and quantify a priori.

When it comes to optic properties, whose knowledge is fundamental for both recreational and professional photography, as well as for photogrammetry, flat and dome ports have their inherent intrinsic pros and cons.

In the following sections, an overview on how the two types of port are addressed in the photogrammetric literature is provided (Section 2.1). Then, a brief description of flat port characteristics is reported (Section 2.2.) with the aim of pointing out the main differences with dome ports. Finally, an extensive and critical analysis on dome ports is provided (Section 2.3).

### 2.1. Underwater Camera Calibration—Literature Review

Flat ports have been intensely studied for photogrammetric underwater applications in relation to camera calibration, an issue that has been faced for almost 50 years [8]. Among the technical and scientific community, underwater photogrammetry is often called multimedia photogrammetry, where the term indicates that the light ray travels across different media: water where the object is immersed, glass or the material the port is made, and air where the camera-lens system works. The transition among these different elements causes a ray’s deviation from the path that it would travel if it were travelling just in air (see Section 2.2). This deviation must be taken into account if a source of error in the measurement process wants to be eliminated. Two main approaches for handling this issue have been proposed in the literature: (i) the collinearity model is modified to take into account the rigorous geometric interpretation of light propagation in multimedia (camera housing-water), also known as ray tracing approach [9]; (ii) the refractive effect of the different interfaces is absorbed by camera calibration parameters using a standard pinhole camera model and a terrestrial-like self-calibration approach [10].

A variety of different methods for a rigorous modelling of underwater image formation has been proposed. Mulsow [11] proposed a multi-media bundle models, where refractive indices surface and even mathematical parameters of the interfaces are introduced as unknowns: the method is particularly suited for Particle Image Velocimetry (PIV) applications and needs to be tested in underwater environment [11]. Jordt-Sedlazeck and Koch [12] propose a light propagation model to be used for color correction along with a strict underwater camera calibration [12]. Treibitz *et al.* [13] include also the distance of the lens from the medium interface to take into account the position variation of the entrance pupil [13]. Agrafiotis and Georgopoulos included the percentages of air and water within the total camera-to-object distance as parameter in their calibration model [14].

The second camera calibration approach is justified by the evidence that the principal component of refractive effects is radial. Therefore, it can be implicitly compensated by the standard, odd-ordered polynomial model for radial distortion, whilst any residual effects from asymmetric components of the housing are partly or wholly absorbed into other parameters of the camera calibration, such as decentering lens distortion or the affinity term [15]. Moreover, the environmental conditions can be hardly modelled *a priori*: refractive index of water changes with depth, temperature and salinity, the shape of the camera housings and port may change with depth due to changing pressure levels [16]. Hence, calibrating the underwater system (camera and lens inside the waterproof case) at the predominant working conditions (depth, temperature, *etc.*) would provide more accurate and reliable results [15].

To avoid severe refraction effects, dome ports can be adopted [17]. In this case, the pinhole camera model would be completely fulfilled if the center of perspective of the camera-lens system were exactly placed in the dome surface center of curvature. In the authors’ knowledge, few studies have tried to quantify the limit of the above statement.

Kunz and Singh [18] proposed a model for hemispherical port calibration to be performed in air and used underwater. The authors simulate the effect of a centering error of entrance pupil in a pressure housing with dome port but they do not provide any theoretical or practical evidence of the employed values [18].

Besides the type of lens port used, the mechanical stability of the whole system camera + lens + underwater housings is an important factor to be considered. Shortis *et al*. [19] investigated the camera-case stability, showing that significant variations in camera calibration parameters are found when removing the camera from the waterproof housing for example to download the images and then reassembling to use the system again straight after [19]. As a concluding remark, a degradation of the geometric accuracy by a factor two in underwater/multimedia photogrammetry should be expected [20].

### 2.2. Flat Lens Port

A flat port is essentially a flat plane of optically transparent glass or plastic in front of the lens, as depicted in Figure 1a–h. Flat ports are the most common waterproof housing of compact digital cameras, being their manufacturing less expensive than dome ports.

**Figure 2:**
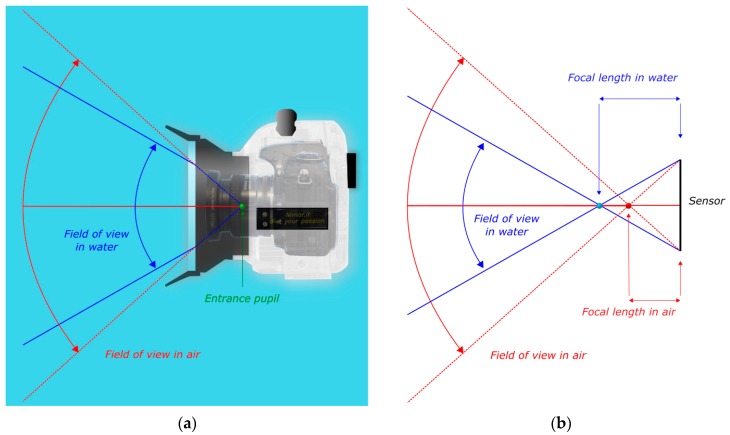
Effect of flat port—Reduction of FOV. (**a**) Optical rays (in red) from the camera when pass through the planar boundary are bent towards the normal to the flat surface, narrowing the field of view; (**b**) This phenomenon can be regarded as an increase of the nominal focal length.

Neglecting the thickness of the port, the flat surface acts as boundary or interface between two different media: water outside the waterproof case and air inside. The two media are characterized by different refractive indices, bigger for the denser water and smaller for air. Consequently, the rays of light coming from object in water deviate from their original path when pass through port and reach the camera sensor. Specifically, each ray is bent toward the normal to the boundary surface according to the Snell’s law. This phenomenon causes that the camera-lens field of view (FOV) is reduced, as shown in in Figure 2. The white box represents a waterproof housing with flat port with inside a Nikon D300 (DX format) mounting a 24 mm lens (Nikon); the cyan part represents the water. For the sake of simplicity, the thickness of the port in the figure is ignored. In red the nominal focal lens and FOV in air are shown (Figure 2b). Because of the presence of flat port, the FOV is narrowed and, conversely, the focal length is increased.

**Figure 3:**
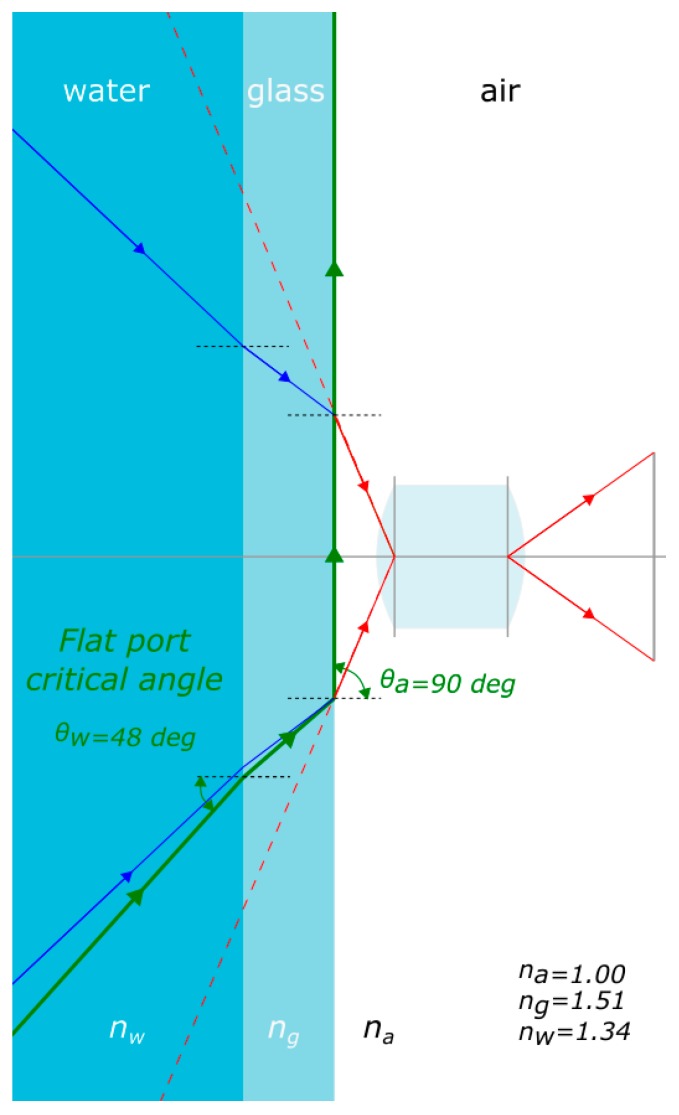
In a flat port the maximum field of view is limited by the total internal reflection.

Flat ports have also a limitation in terms of maximum field of view. Indeed a ray of light entering the glass of the port from water and then living the interface glass/air is subject to total internal reflection as the ray passes from a means with higher refraction index to another with lower one. By applying Snell’s law, the critical angle can be calculated, leading to a maximum field of view for every flat port of about 96° as visible from Figure 3 where, as the entrance angle $\theta_{w}$ rises, the refracted angle is bent away from the normal to the flat port surface. When it reaches about 48° the ray does not enter the housing anymore as it is subject to total internal reflection (green ray). As expected the angle of refraction $\theta_{a}\;$is 90° in this case.

As one would expect, by lowering the FOV and keeping the subject at the same distance from the camera, the magnification of the object becomes larger than in air by a factor approximately equal to the ratio between the refraction indices of water and air. Moreover the object appears closer to the camera. When a flat port is used, not only the FOV and focal length vary, but also the lens distortion is affected.

**Figure 4:**
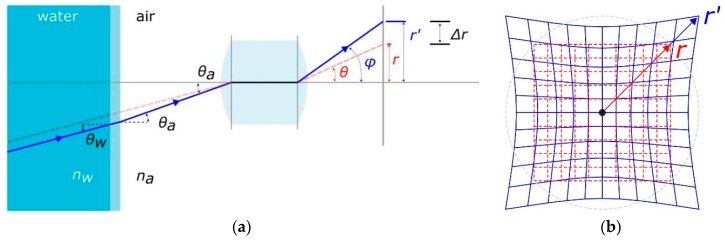
Effect of a flat port on image distortion: (**a**) section view; (**b**) image plane view.

Typically, a ray of light passing from water through a flat port introduces a pincushion radial symmetric distortion as depicted in Figure 4. The objective lens inside the housing outlined in the figure is supposed to be free from any type of geometric distortions and would reproduce an undistorted image of an object when placed in air. Conversely, placing the camera underwater, in a pressure housing with flat port, would produce a prominent pincushion distortion depicted in blue in Figure 4b. Under the assumption that the distance between the lens and flat port can be neglected, the image distortion factor *D*, expressed ad the ratio between *r′/r*, can be computed using the following formula [21]:

(1) $$D={\left(\frac{n_{w}^{2}-sin^{2}\;\theta_{a}}{1-sin^{2}\;\theta_{a}}\right)}^{1/2}$$

where:

$\theta_{a}$ is the entrance angle in air;
$n_{w}$ is the refraction coefficient of water.

In addition to radial distortion, flat lens port also introduces chromatic aberration. The rays of light, when refracted, are separated into different wavelengths of the visible color spectrum (component colors) that do not travel at the same speed and when passing through the flat port are differently bent. The separate components can overlap, causing a loss of sharpness and color saturation.

### 2.3. Spherical or Hemispherical Dome Lens Port

**Figure 5:**
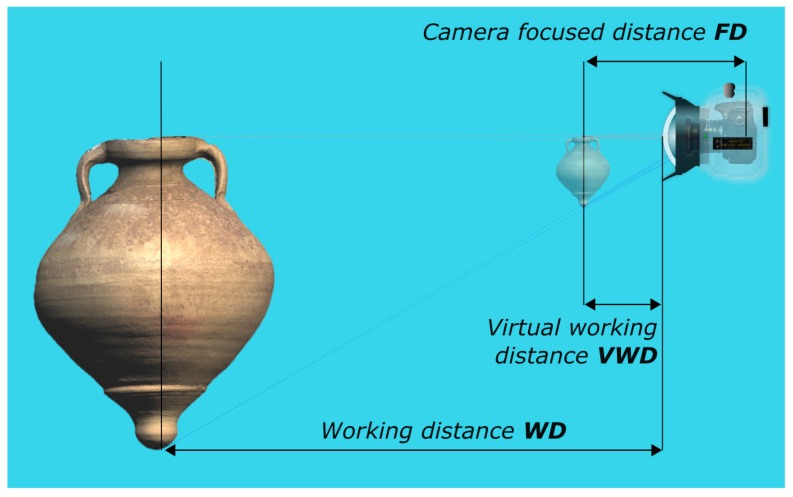
Effect of a dome port: a subject located at a working distance (WD) appears smaller and much closer to the camera—virtual working distance (VWD).

Spherical or hemispherical dome lens port, like the one shown in Figure 1i, solves the problems introduced by flat port, *i.e*., FOV and focal length of the camera-lens system are preserved, peculiarity very crucial when dealing with wide angle lenses. Nevertheless, other issues arise when using dome port. A spherical dome port is a concentric lens that acts as an additional optical element to the camera lens. Indeed, it is a real lens, more precisely, a negative or diverging lens: both the focal length and image distance are negative so that the image is formed to the left, *i.e*., in front of the dome. Such an image is called virtual image and is upright and smaller than the object (Figure 5—produced using the free optical design software WinLens 3D Basic by Qioptiq). The camera-lens system behind the dome port will actually focus not on the real object but on the virtual image produced by the dome port itself at a smaller distance than the object. 

Being the dome port a spherical lens, it suffers from spherical aberrations *i.e.*, optical rays passing through the peripheral parts of the dome do not converge in the same focal point of the rays passing through the center. The result is that there is not a single image plane and a blurred image can be produced.

**Figure 6:**
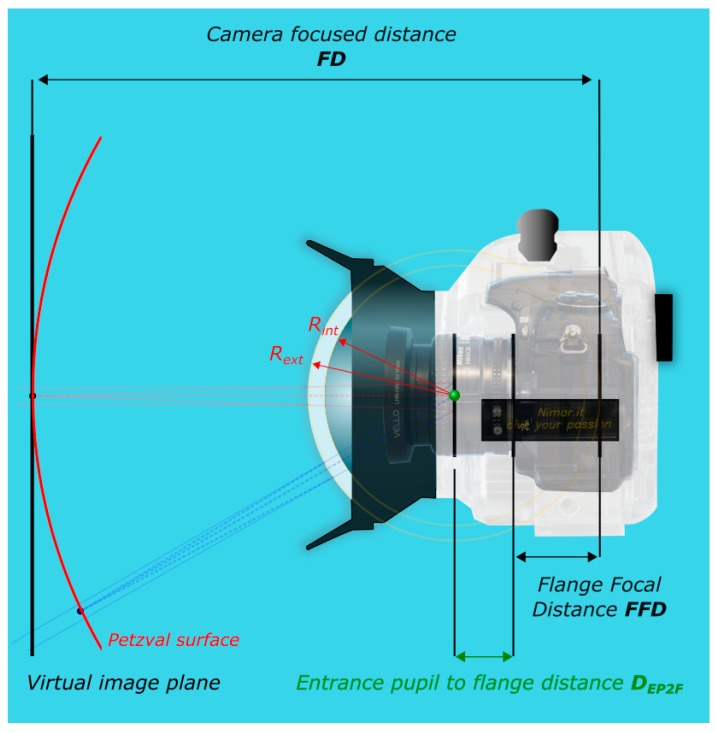
Main photographic elements in the image formation through a dome port.

Another undesirable optical effect of a spherical dome port is the field curvature that causes flat object to be projected on a paraboloidal surface, known as Petzval surface (Figure 6). Being instead the image sensor flat, the consequence is that the object can appear not completely in focus or not uniformly sharp across the image.

In summary, differently from flat lens ports, dome ports:

1. preserve the FOV and focal length of the camera-lens system;
2. do not change significantly the shape of lens distortion;
3. cause the camera-lens system to focus much closer than in air;
4. can introduce spherical aberrations and field curvature, producing unsharp images.
5. increase the DOF.

Points 1 and 2 are completely verified if the entrance pupil of the camera lens coincides with the center of curvature of the dome port (Figure 5). The larger the distance between entrance pupil and center of curvature, the greater the geometric distortions and chromatic aberrations introduced.

The effect of the non-concentricity of the dome port and entrance pupil can be easily simulated using Winlens software. The behavior of the misalignment can be summarized as follows:

(1) a misalignment of the dome port on a plane orthogonal to the optical axis of the objective lens produces decentering distortions;
(2) a misalignment along the optical axis of the objective lens produces a pincushion-type radial symmetric distortion if the center of the pupil entrance is in front of the center of the spherical dome whereas a barrel-type radial symmetric distortion if behind.

Despite being a paramount point for photographers and photogrammetrists, especially for spherical photogrammetry applications, the position of the center of the entrance pupil is not provided by lens manufacturer. Its position changes when focusing and it is not easy to predict its motion as it depends on the optical design of the lens, most of the times only partially available. An objective lens is a system composed of many lenses through which the bundle of rays, entering the objective, converge and diverge in their passage. The amount of rays which enter the lens is controlled by a diaphragm known as aperture stop. The entrance pupil represents the center of perspective of the lens and can be seen as the virtual image of the hole materialized by the aperture stop seen from the front of the lens [21]. When placed inside the pressure housing, behind a spherical dome port whose radius is R, the virtual image of an object at infinity will be projected in front of the dome at about 4R from the center of the spherical dome or 3R from the dome glass. For a dome port of 8 cm radius the real world from infinity up to 1 m from the dome surface is compressed in a virtual space in front of the dome which extends approximately from 17 cm to 23 cm from the glass (see Section 4). It is clear that the camera must be able to focus at these distances to obtain a sharp image. The popular Gopro Hero sport camera (up to the version 2) suffered from out-of-focus images when placed underwater because of the small radius of its dome port and impossibility to focus on the virtual image due to fixed focus. This was a big issue for such a compact camera camera so that in the newer version a flat port was adopted thus reducing the field of view.

## 3. Laboratory Investigation and Geometric Characterization of NiMAR NI320 Dome Port and NI303D Waterproof Case

**Figure 7:**
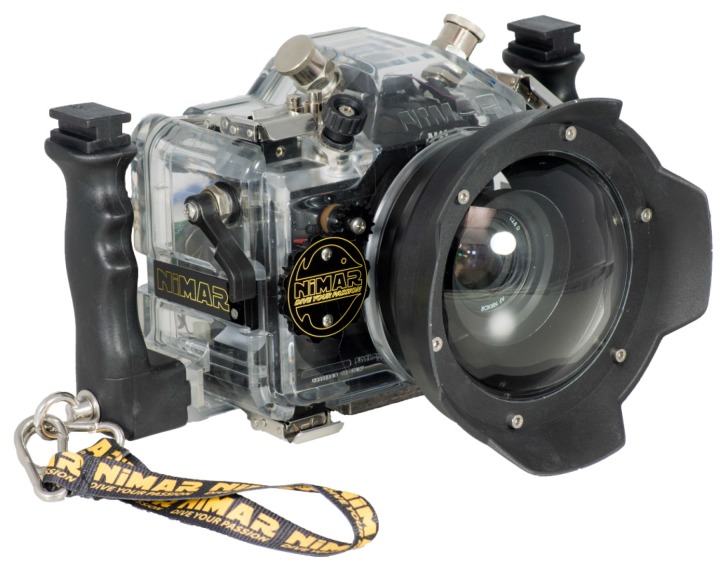
NiMAR NI320 dome port and NI303D waterproof case.

The aim of this investigation is to study the manufacturing of a commercial underwater housing with dome port (Figure 7), *i.e*., to measure the following fundamental characteristics:

- position of the camera lens entrance pupil (Section 3.1);
- deviation of external and internal dome surfaces from an ideal spherical shell;
- misalignment between the center of curvature of the dome surfaces and entrance pupilof the camera lens.

The second analysis implies the evaluation of the radius and center of curvature of the two spheres fitting the external and internal dome surfaces respectively (Section 3.2).

**Figure 8:**
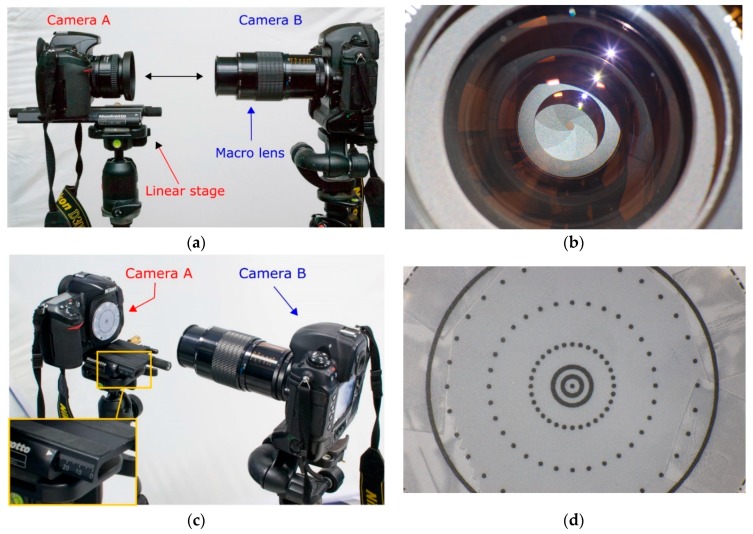
Measurement of the distance D_EP2F_ between the entrance pupil and the flange.

The third requires a more difficult measurement process, described in Section 3.3 and Section 3.4. All the measurements reported in the following parts are referred to the center of the camera flange (Figure 8). Table 1 summarizes the characteristics of the dome port as provided by the manufacturer.

**Table 1:** Manufacturing parameters of the tested NiMAR hemispherical dome.

Product Code	Material	Nikkor Lenses That Can be Fitted in (mm)	Internal Diameter (mm)	External Diameter (mm)	Length (mm)	Weight (gr)
NI320	Crystal glass	20–24–28–35	94	176	91	671

It is worth to note that the same dome port is used with different lenses whose focal length varies from 20 mm to 35 mm. Consequently, also the position of the entrance pupil may vary inside the case. Moreover, the datasheet reports the dimensions of the black frame of the dome whereas no information about the radius of curvature, the center and thickness of the glass surface is usually available.

To recover the information needed to fully characterize the optics of the camera-lens and underwater case-dome systems, a reverse engineering process is carried out through the steps described in following subsections.

### 3.1. Measurement of Lens Entrance Pupil

The distance of the entrance pupil from the flange on the camera body was measured according to the following procedure:

(1) the objective lens is mounted on the camera and placed on a linear stage on a stable tripod which is then leveled accurately, this camera will be called camera *A* (Figure 8a);
(2) another camera *B* with a macro lens is placed on another tripod in front of camera *A,* aligned and leveled accurately to make the optical axes of cameras *A* and *B* as more collinear as possible (Figure 8a);
(3) the aperture stop of camera A is stopped down to close the iris (through for example bulb function or long exposure);
(4) the macro lens on camera *B* is used as collimator and focused on the iris of the objective of camera *A* (Figure 8b)*;*
(5) the lens from camera *A* is removed and substituted with a flat and thin sheet with markers printed on it that will cover the flange of the camera body. The sheet is aligned and attached to the flange of the camera body *A* using thin double sided tape;
(6) the camera body *A* without lens is translated ahead toward the macro lens through the linear stage (Figure 8c) until the markers appears in focus (Figure 8d). Due to the limited depth of field of the camera *B* (macro), the repeatability of linear translation of camera body *A* is in the order of some tenths of millimeter;
(7) the linear translation is taken as offset (distance along the optical axis) of the entrance pupil from the camera flange.

In order to guarantee a better stability of the setup, the tripods were fixed with hot glue on the floor while to improve the readings of linear translations a caliper was used. The measurements were carried out for two lenses, the Nikkor 24 mm f/2.8 AF-D and the Nikkor 35 mm f/2.0 AF-D.

The entrance pupil distance from the flange, here indicated as D_EP2F_, was measured with the lens focused at different distances (focus distance, FD). The subject to entrance pupil distance (D_S2EP_) was also computed according to the following equation:

(2) $$D_{S2EP}=FD-FFD-D_{EP2F}$$

where:

- FD is the focus distance read on the lens barrel and it indicates the distance between the camera sensor and subject;
- FFD is the flange focal distance, equal to 46.5 mm for Nikon cameras;
- D_EP2F_ is the entrance pupil offset measured according to procedure described above.

Table 2 summarizes the computed distances.

**Table 2:** Measured distances between flange and entrance pupil distances.

Lens	FD @ Infinity	FD @ 300 mm		FD @ 250 mm	
	D_EP2F_	D_EP2F_	D_S2EP_	D_EP2F_	D_S2EP_
Nikkor 24 mm f/2.8 AF-D	24.6 mm	26.9 mm	226.6 mm	not possible	not possible
Nikkor 35 mm f/2.0 AF-D	18.6 mm	25.4 mm	228.1 mm	26.9 mm	176.6 mm

The method here described allows the entrance pupil to be precisely located along the optical axis (Z component), but does not provide any information about potential in-plane offsets (XY components, *i.e.*, its position projected onto the flange plane).

The position of entrance pupil was measured also for the lens focused at infinity for the sake of completeness. However, it is worth to note that with a dome of radius comparable to the ones under investigation the virtual image of a subject very far away from the camera (≈@ infinity) is formed at a distance less than 290 mm from the entrance pupil. As a consequence, in such a condition, the camera focus should not be set to infinity to avoid blurred or out of focus images.

### 3.2. 3D Model of NI320 Dome Port

**Figure 9:**
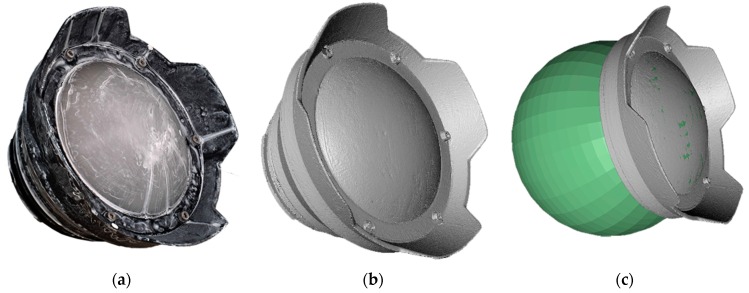
Reverse engineering of NI320 dome port: (**a**) opaque coating applied to avoid reflection and wrong measurements; (**b**) obtained polygonal model; (**c**) fitting of a sphere on the external dome surface.

An opaque coating was applied over the transparent glass dome NI320 (Figure 9a), which was surveyed using a triangulation based laser scanner (ShapeGrabber, Ottawa, ON, Canada [22]). The produced polygonal model, shown in Figure 9b, has a surface sampling step of about 0.25 mm.

Two spheres are fitted to the external (Figure 9c) and internal dome surfaces, respectively (Table 3), showing that the two centers of curvature do not coincide but have a distance less than 1.5 mm. The actual thickness of the spherical dome is computed as difference between the two radii and it is equal to 7.7 mm.

**Table 3:** Fitting of external and internal dome surfaces.

	Fit Statistics		Sphere			
	Num. of Points	Stdv (mm)	Centre (mm)			Radius (mm)
			X	Y	Z	
External	≈170,000	>0.1	−0.4	−1.0	21.5	83.4
Internal	≈150,000	>0.1	−0.4	−1.5	20.3	75.7

### 3.3. Measurement of the Nikon D300 Camera and NI303D Pressure Housing Flange Centers

The flange is a metal ring on digital cameras and a plastic ring on underwater cases where the rear of the lens and the rear of the flat/dome port are respectively mounted. The position of the camera mounting flange with respect to the waterproof housing is fundamental in order to locate the lens entrance pupil (see Section 3.1). Analogously, the identification of the plane containing the housing flange is needed to find the position of the centers of dome surfaces relatively to the camera-lens + housing-port system.

**Figure 10:**
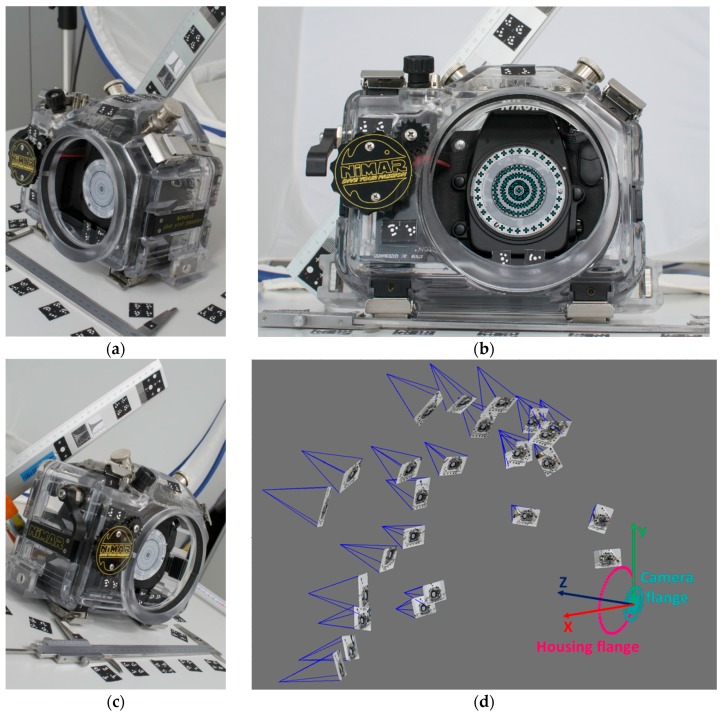
Photogrammetric survey of camera and pressure case flanges: (**a**) and (**b**) examples of acquired images; (**c**) dots identifying the camera flange markers in an image; (**d**) camera network and measured 3D points for the identification of camera and housing flanges with the established reference system.

To find the relative position between the two mounting flanges, *i.e*., for the camera and underwater case, a photogrammetric survey was conducted (Figure 10). The two planes containing the elements of interest were materialized and identified through black and white dots measured in the images (Figure 10c). The target positions on the circular sheet attached to the camera flange were designed to be concentric with the Nikon F mount flange. The estimated centering error is less than 0.5 mm. The 3D coordinates of the triangulated dots were fitted through a least squares procedure to find the reference planes and centers of the flange for both camera and housing.

The coordinate measurement system was fixed in the center of the camera flange, with the XY plane coincident with the camera flange plane, and the Z axis along the optical axis, positive toward the housing flange (Figure 10d).

### 3.4. Assembly of the Different Measurements

**Figure 11:**
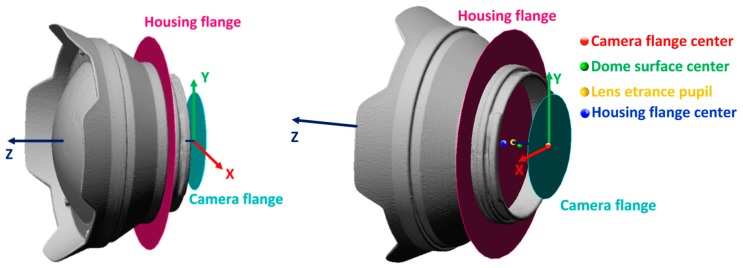
Two views of NI303D housing with NI320 dome port and reference planes and points.

A plane was fitted to the rear of the dome port and aligned to the flange plane of the housing (magenta plane in Figure 11). From the measurements described in the previous steps, the relative position of key geometric and optic elements is known and the complete geometry of the camera-lens + housing-dome system is reconstructed.

The main outcome of the survey described above is that, in the worst condition, the position of the entrance pupil both for the 24 mm and 35 mm lens results maximum 5.4 mm head of the center of curvature of the dome surfaces (*i.e.*, closer to the dome), while the misalignment in the XY plane, due to the misalignment of the dome center of curvature, is about 1 mm (Table 4).

**Table 4:** Coordinates of key geometric and optic elements.

Point Name	Position (mm)		
	X	Y	Z
Camera flange center	0.0	0.0	0.0
Dome surface center	−0.4	−1.0	21.5
Lens entrance pupil	0	0	26.9
Housing flange center	−0.4	−1.0	34.3

## 4. Optic Characterization

Using the data of the NI320 dome port obtained through the reverse engineering process described in Section 3, an optic characterization of the same view port is carried out using the optical ray tracing software Winlens. Using the values of the internal and external radius (Table 3) and the thickness computed as difference between the external and internal radii, under paraxial assumptions, positions of the virtual image *versus* the real object can be computed. The dome port glass is a N-BK7, very commonly used for underwater view port and characterized by a refraction coefficient of 1.5168.

Table 5 lists the values of working distances (*WD*) from a real object point underwater *versus* its virtual image distance (or subject to entrance pupil distance *D_S2EP_*) from the entrance pupil (supposed to be placed in the dome center). Virtual working distances (VWD), as described in Figure 5, are also reported. For the sake of simplicity the distances are here considered positive, even if for convention they should be negative quantities.

**Table 5:** Real object distance *versus* its virtual image underwater for the NiMAR NI320 dome port.

*WD* (mm)	200	300	400	500	750	1000	3000	5000	10000	Infinity
*D_S2EP_* (mm)	164.1	184.9	199.7	210.8	229.4	240.8	269.7	276.8	282.5	288.5
VWD (mm)	80.8	101.6	116.4	127.5	146.1	157.5	186.4	193.5	199.2	205.2

**Figure 12:**
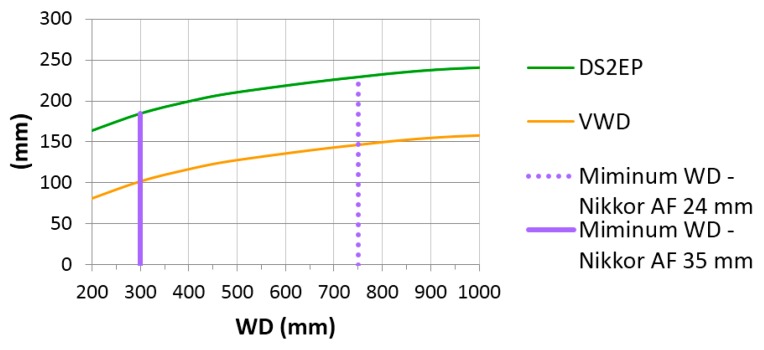
Variation of subject to entrance pupil distance (DS2EP) and virtual working distance (VWD) in function of the working distance (WD). The minimum WD for Nikkor 24 mm and 35 mm focal length are also drawn.

In Table 5 it is evident how the virtual image of a real object underwater is compressed in a very narrow virtual space just 20 cm deep in front of the dome glass. By using the values of *D_S2EP_* from Table 2 corresponding to minimum focusing distances for the two Nikkor AF 24 and 35 mm lenses and closest corresponding values from Table 5, the minimum working distances WD for the two lenses result to be respectively −750 mm and −300 mm (Figure 12). For closer objects, additional close up lenses must be mounted to the front of the camera in order to produce sharp images.

### Underwater Variation of the DOF

This section presents a simulation which demonstrates and quantifies the increase of DOF underwater, one of the main characteristics of dome ports introduced in Section 2.3. The following analysis is performed, under paraxial assumptions, for an aperture value f/8 and a circle of confusion of 15 microns (<3× pixel size). Let us consider a Nikon D300 with 24 mm lens in the NiMAR NI303D housing with the NI320 dome port and the subject to be at 1 m distance from the entrance pupil of the 24 mm lens. In this computation, the entrance pupil is supposed to be concentric to the spherical dome. Under these hypotheses, when underwater, a virtual image from the entrance pupil is formed at 238 mm, on which the camera has to be focused. Near and far sharp limits (NSL and FSL, respectively) values of the DOF result equal to −227 mm and −249 mm. These values in the virtual image space correspond to planes at −792 mm and −1355 mm distances from the camera in the real object space, leading to a total DOF of 563 mm. In air, for the same set up and the object at a 1 m distance from the entrance pupil, the total DOF would be 424 mm, with the NSL and FSL respectively at −831 mm and −1260 mm., The ratio between the DOF in water and in air approaches the ratio between the two refraction indexes, *i.e*., 1.33–1.34 which corresponds to a relative increase of about 33%–34% of the DOF underwater.

## 5. Swimming Pool Tests

In the previous sections, the optic characteristics of dome ports have been described from a theoretical point of view and then proved with a real case example. In the followings, the influence of the view port on the photogrammetric system (camera + lens) is quantified in a real underwater scenario through some tests performed in a swimming pool. The results afterwards presented are part of some tests aimed at investigating the performance of consumer-grade underwater camera housings when used for photogrammetric purposes.

### Camera Calibration

**Figure 13:**
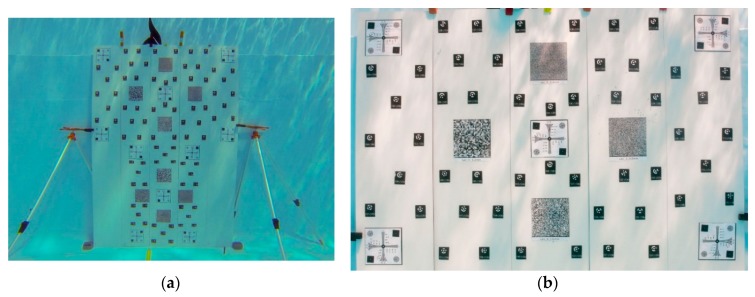
Test-field used in the swimming pool: (**a**) overall view of the complete board with stands; (**b**) part of the board used for the camera calibration.

A first prototype of an underwater test-field made of a planar aluminum board was temporarily fixed on a wall of the pool (Figure 13a). The test-field was prepared with photogrammetric coded targets and some resolution targets (Figure 13b).

Two photogrammetric acquisitions for self-calibration were realized, one underwater and one in air. The camera with the 24 mm at f/8.0 was focused underwater at 1m using the autofocus system then the autofocus was disabled to keep the interior orientation parameters of the camera stable as much as possible. Between the two image acquisitions, the camera was not removed from the pressure housing to keep the assembly as much stable as possible. Table 6 reports the camera calibration parameters obtained from the two calibrations.

**Table 6:** Comparison between camera calibration in water (UW) and in air. Some non-significant additional parameters were not computed during the self-calibration procedure.

Camera Calibration Parameters	AIR		UW	
	value	std	value	std
Principal distance (mm)	25.801	0.006	26.208	0.002
Principal Point x_0_ (mm)	−0.026	0.002	−0.058	0.003
Principal Point y_0_ (mm)	−0.144	0.003	−0.207	0.002
K1	1.842e-004	1.2e-006	1.663e-004	6.1e-007
K2	−3.030e-007	7.4e-009	−2.582e-007	3.4e-009
K3	-	-	-	-
P1	-	-	6.582e-006	1.2e-006
P2	-	-	1.620e-005	8.7e-007

**Figure 14:**
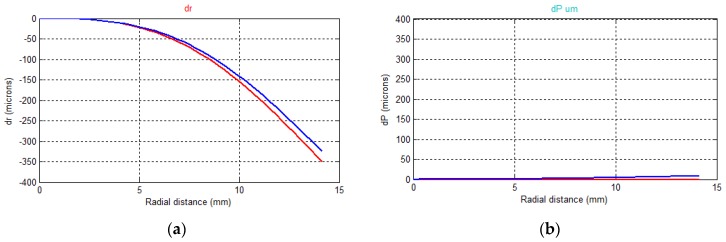
(**a**) Radial and (**b**) decentering distortion curves: the curves in red are related to the camera calibration in air, the curves in blue to the camera calibration underwater.

As shown in Figure 14, the lens displays quite a pronounced barrel radial distortion both in air (red) and in water (blue). As previously anticipated by the reverse engineering of the dome, the advanced position of the entrance pupil of the lens respect to the dome center introduces a small pincushion compensation effect resulting in a less negative overall distortion (less barrel). A significant variation in the principal distance between in air and underwater calibrations is also observed. This change is expected as the closer is the lens to the dome surface, the less spherical is the portion of the surface of the dome the camera looks trough. The extreme limit is when the lens front is very close to the dome inner surface and the entrance pupil is much more ahead than in the case study of this paper: in this case the dome portion in the field of view of the camera approaches the one of a flat port with a consequent increase of the principal distance by a factor of about 1.33 as explained in Section 2.2.

Decentering distortion is introduced, due to the in-plane offset between lens entrance pupil and dome surface center. In air the decentering distortion parameters were not statistically significant thus were not adjusted for. As it can be observed in the graph, its magnitude in water is anyway very small compared to the radial component, as expected due to the smaller in-plane than along the axis misalignment.

The in-plane offset can also explain the difference in the coordinates of the principal points.

**Figure 15:**
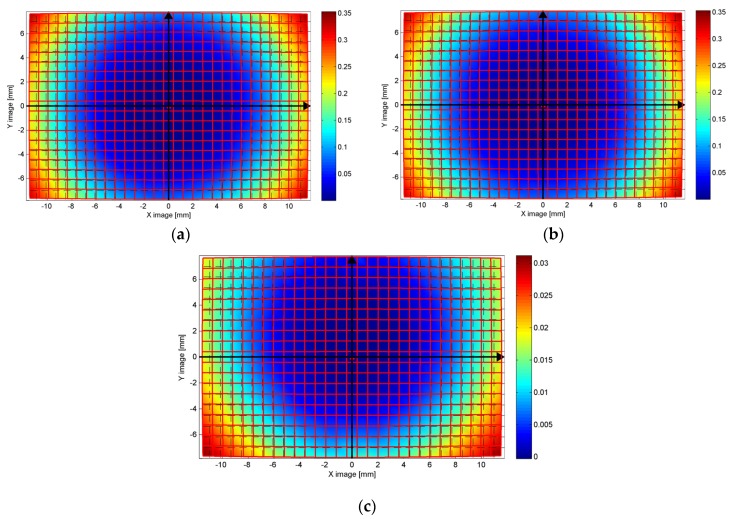
Distortion maps (difference in mm between ideal and actual distorted pixel position): (**a**) in air, (**b**) in water; and (**c**) difference air-water.

**Figure 16:**
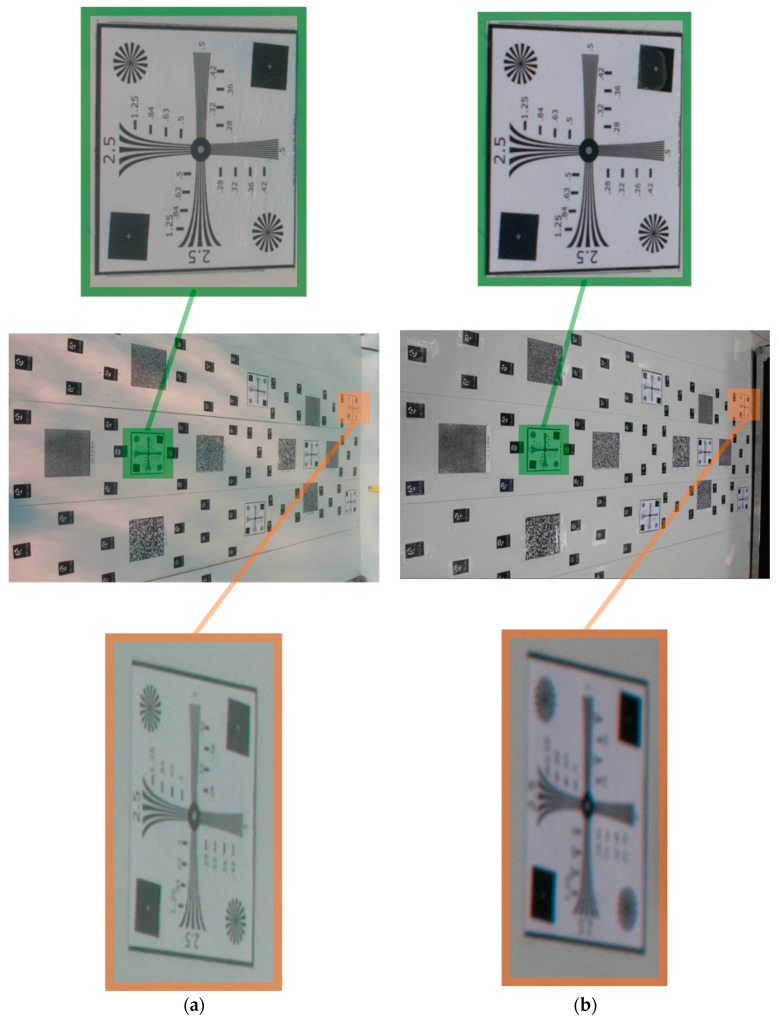
DOF variation between two pictures taken from very similar positions (**a**) underwater and (**b**) in air.

In Figure 15, the system distortions are visualized according to a color map (distortion map): the color represents the difference between the ideal pixel position (no distortion) and the actual pixel position due to the influence of radial and decentering distortions determined through camera calibration. The difference between the distortion map in air (Figure 15a) and in water (Figure 15b) is reported in Figure 15c. As expected, the maximum difference is reached at the borders, whose magnitude is comparable with the differences highlighted in the distortion curves. An asymmetric behavior can be also observed, likely due to the small in-plane misalignment between the lens entrance pupil and dome surface center of curvature, slightly bigger along the Y axis. The optic behavior of the dome port results to be well modelled by the pinhole camera and Brown’s distortion model. It is worth to note that the experiments were carried out at a small depth; consequently, it may be expected that the influence of the pressure on the watertight case is considerably less critical and probably easier to be absorbed by standard calibration parameters than in deep water. To demonstrate the DOF increasing in underwater situations, a slant view of the test-field is analysed (Figure 16). An image used during the underwater calibration was compared with a very similar one acquired in the laboratory. Both cameras are in the pressure housing and focused so that an object is sharp at 1m from the entrance pupil of the lens.

As visible in Figure 16 the increase of DOF underwater is quite evident and in accordance with what anticipated in Section 4. Indeed, the resolution target highlighted in green was at about 1m while the orange one was at 1.75 m from the camera. Whilst the target at 1 m is sharp in both the images taken underwater and in air, the one at 1.75 m from the camera is completely out of focus in the image taken in air.

## 6. Conclusions and Future Developments

The paper presented the optic and geometric characterization of a consumer grade pressure camera housing (manufactured by NiMAR) that was successfully used in the underwater survey of the Costa Concordia gash [6]. The main advantages and drawbacks of flat and dome glasses were presented, in particular with respect to parameters used in the photogrammetric planning. Indeed, the concepts of virtual images generated by dome ports are often difficult to understand, especially for non-experts in surveying and photogrammetry and may lead to wrong or not optimized practices in the field (*i.e.*, focus fixed at infinity or pre-focused in air or too high aperture values for increasing the DOF). The 3D reverse engineering of a NiMAR dome port and its position relatively to the entrance pupil of the lens served as input for some computer simulations carried out through a freely available optical ray tracing software. The computer analyses anticipated some behaviors on the camera calibration parameters concerning the radial and decentering distortions when the entrance pupil is ahead or behind, or laterally displaced to the center of curvature of the dome. A DOF increasing in underwater scenarios was also anticipated trough computer analyses.

The final swimming pool tests sustained and demonstrated the validity of computer simulations. These tests will be soon followed by other analyses and tests underwater in swimming pool and in open water to deliver photogrammetric guidelines for underwater camera housing and applications.

**Figure 17:**
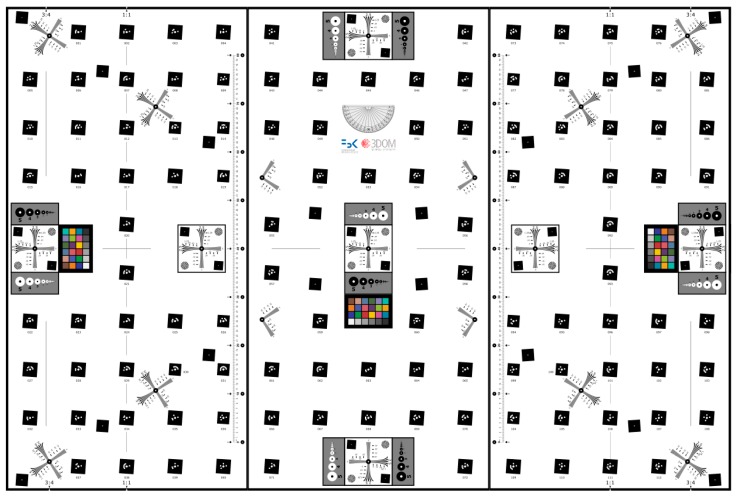
The newly designed modular test-field for resolution and DOF measurements as well as camera calibration. The test-field, being modular, can be mounted to accommodate different heights of the targets.

The developed tests served to design a new modular calibration test-field whose base plane is shown in Figure 17. The test-field displays circular coded target on a slant square background for MTF measurements, DOF evaluation and geometric camera calibration. Some of the targets will be stuck on out-of-plane elements to make the test-field more suitable for camera calibration (depth variation).
